# 943. Zeroing In: Targeted Bundle Eliminates MRSA Bloodstream Infections and Pneumonias

**DOI:** 10.1093/jbcr/irag033.510

**Published:** 2026-04-06

**Authors:** Laura E Carpenter, Morgan Santos, Colleen A Jennings, Sharon A Taylor, Joshua Schuster, Tracy Larson

**Affiliations:** Warden Burn Center at Orlando Health Orlando Regional Medical Center, Orlando, FL; Warden Burn Center at Orlando Health Orlando Regional Medical Center, Orlando, FL; Warden Burn Center at Orlando Health Orlando Regional Medical Center, Orlando, FL; Warden Burn Center at Orlando Health Orlando Regional Medical Center, Orlando, FL; Warden Burn Center at Orlando Health Orlando Regional Medical Center, Orlando, FL; Orlando Health, Daytona Beach, FL

## Abstract

**Introduction:**

Hospital-onset MRSA bloodstream infections affect up to 21% of burn ICU patients. Despite national efforts, the U.S. has only achieved a 25% reduction in hospital-onset MRSA bacteremia from its 2015 baseline—falling short of the 50% national goal. These infections are linked to increased mortality, longer hospital stays, and higher healthcare costs. In early 2025, the Burn Trauma ICU (BTICU) saw a statistically significant rise in hospital-onset MRSA BSIs, despite historically low rates. In response, a multidisciplinary team was formed to investigate contributing factors and implement targeted prevention strategies.

**Methods:**

A retrospective analysis of BTICU patients admitted in 2025 evaluated the effectiveness of targeted infection prevention strategies. A multidisciplinary team—nursing leadership, staff nurses, respiratory therapists, infection preventionists, hospital administrators, physician leaders, and clinical quality experts—reviewed infection control practices, workflows, and environmental hygiene protocols to identify improvement opportunities and enhance patient safety. A targeted MRSA prevention bundle was developed and implemented, including:

• Manual ventilator cuff pressure monitoring, compared to continuous systems, to reduce ventilator-associated pneumonia.

• Enhanced oral care protocols to improve adherence and reduce oropharyngeal colonization.

• Intranasal mupirocin replaced povidone-iodine for more effective MRSA decolonization.

Additional measures included daily chlorhexidine bathing, reinforced hand and glove hygiene, 18-point cleaning of high-touch surfaces, UV terminal disinfection, and weekly central line, room, and bed changes for burn patients. These interventions were supported through staff education, compliance monitoring, and interdisciplinary collaboration.

**Results:**

Prior to the intervention, eight patients were reviewed, five of whom were burn patients. Among these, seven MRSA BSIs and six MRSA pneumonias were identified between January and May 2025.

Following the implementation of the MRSA prevention bundle and enhanced infection control protocols, the BTICU reported zero MRSA BSIs and zero MRSA pneumonias in June and July 2025. The most notable improvement was the elimination of MRSA ventilator-associated pneumonias, which dropped from six cases in the first five months of the year to zero by September.

**Conclusions:**

The integration of manual cuff pressure monitoring, improved nasal decolonization practices, and increased oral care compliance contributed to a marked reduction in MRSA-related infections. These findings underscore the importance of targeted, evidence-based infection prevention strategies in high-risk units.

**Applicability of Research to Practice:**

This initiative applies evidence-based strategies to reduce MRSA transmission and improve outcomes; further research is needed to assess individual bundle components’ effectiveness in preventing BSIs.

**Funding for the study:**

N/A.

